# Transparent and tough bulk composites inspired by nacre

**DOI:** 10.1038/s41467-019-10829-2

**Published:** 2019-06-26

**Authors:** Tommaso Magrini, Florian Bouville, Alessandro Lauria, Hortense Le Ferrand, Tobias P. Niebel, André R. Studart

**Affiliations:** 1 Complex Materials, Department of Materials, ETH Zurich, Zurich, 8093 Switzerland; 2 Multifunctional Materials, Department of Materials, ETH Zurich, Zurich, 8093 Switzerland; 3 Present Address: Imperial College London, Department of Materials, London, SW7 2AZ UK

**Keywords:** Bioinspired materials, Composites, Mechanical properties

## Abstract

Materials combining optical transparency and mechanical strength are highly demanded for electronic displays, structural windows and in the arts, but the oxide-based glasses currently used in most of these applications suffer from brittle fracture and low crack tolerance. We report a simple approach to fabricate bulk transparent materials with a nacre-like architecture that can effectively arrest the propagation of cracks during fracture. Mechanical characterization shows that our glass-based composites exceed up to a factor of 3 the fracture toughness of common glasses, while keeping flexural strengths comparable to transparent polymers, silica- and soda-lime glasses. Due to the presence of stiff reinforcing platelets, the hardness of the obtained composites is an order of magnitude higher than that of transparent polymers. By implementing biological design principles into glass-based materials at the microscale, our approach opens a promising new avenue for the manufacturing of structural materials combining antagonistic functional properties.

## Abstract (web summary)

Transparent materials with high strength and hardness coupled with low crack tolerance remain challenging to manufacture. Here, the authors develop a process to fabricate transparent but tough glass composites with a nacre-like architecture that slows crack propagation.

## Introduction

Optical transparency is ubiquitous in modern technologies. This is evident from the numerous devices and components we interact with in everyday life, from glasses in electronic displays to vehicle windshields and large-area windows in buildings. Besides optical transparency, all these applications require materials that are sufficiently strong and hard to withstand the wearing and high mechanical stresses experienced in use. This set of requirements has made silica-based glasses the primary choice for such applications. However, silica-based glasses are still prone to shattering and catastrophic failure. These events not only alter the structural integrity of the glass parts but also affect their reliability under service. In contrast to silica-based glasses, transparent polymers, such as polyacrylic, polystyrene, and polycarbonate-based materials, are inexpensive, easily processed and shaped, but lack the hardness to resist wear over long periods of use. Therefore, the development of bulk materials that combine high strength, toughness, and wear resistance, while keeping optical transparency, is crucial for the realization of the next generation of electronic devices and structural components.

Glass mainly fails when tensile stress states concentrate in the presence of defects. Defects can be introduced on the glass surfaces either during the manufacture process or under service, due to impacts, wear, and scratching. Current approaches to increase the resistance against fracture of glass often rely on the introduction of compressive stress states on the outermost surfaces, mainly by thermal and chemical treatments, resulting in the so-called tempered glasses^1–3^. This typically leads to a four- to sixfold increase in the mechanical strength, compared with untreated silica glass, and conversely to a substantial increase in the resistance against crack initiation. Despite the resulting strengthening effect, thermal and chemical treatments do not introduce any toughening mechanism in the glass parts, leaving unaltered the already poor resistance against crack propagation in the material. Indeed, once crack propagation is initiated, even tempered glasses do not show any further resistance against fracture. For this reason, accurate predictions on the loading conditions, alongside the replacement of damaged parts (as it happens with scratched screen covers) are crucial aspects to take into account to avoid unpredictable and catastrophic fractures, that impede the structural reliability of the parts. To ensure safety in structural applications, bulk glass is often combined with thin polymer sheets to form laminates that prevent the spreading of debris generated upon catastrophic fracture^4,5^.

In contrast to glass tempering, microstructuring through laser engraving has recently been shown to be an effective approach to imbue brittle glasses with true fracture-toughening mechanisms^6^. In such process, a laser is used to create weak architectured interfaces that deflect cracks and provide some of the energy-dissipating mechanisms, often found in tough biological materials, like teeth and mollusk shells. This bioinspired approach increases the toughness of the material by two orders of magnitude compared with standard brittle glass. Such toughening is, however, accompanied by a tenfold decrease in fracture strength due to the relatively large microstructural defects intentionally created by the laser, which are typically tens of micrometers in size^6^. Hence, glass-based architectured materials with microstructural features at the submicron scale are expected to show high toughness without compromising their intrinsically high transparency and mechanical strength.

Designing architectures with energy-dissipating structural features at the submicron scale is a strategy often used by living organisms to create biological materials that combine fracture toughness and optical transparency using inherently brittle building blocks. Chiton, starfish, and even mollusk shells follow this approach to grow highly mineralized exoskeletons that are both transparent and mechanically robust^7–11^. In the shell of the mollusk *Placuna placenta*, for example, the internal arrangement of brittle calcium carbonate microplatelets into a brick-and-mortar architecture and the presence of organic and ionic inclusions enable a twofold increase in hardness when compared with calcium carbonate monocrystals^10^. Although conventional fracture resistance measurements could not be performed due to the limited size of the specimen, the *P. placenta* seashell shows a much more damage-tolerant response against micromechanical indentation than the calcium carbonate monocrystals^10,11^. Other mollusk seashells with similar but less mineralized structure, such as the shell of the *Haliotis rufescens*, show fracture toughness that is up to a 40-fold higher than their equivalent calcium carbonate monocrystals^12,13^ without, however, being optically transparent. The remarkable toughening effect of biological brick-and-mortar structures results from submicron structural features that promote crack deflection, bridging, viscoplastic dissipation, platelet pullout, and interlocking mechanisms programmed within the material’s architecture^13–15^. From the several energy-dissipating mechanisms that are activated during fracture of such nacreous materials, the combination of highly aligned inorganic platelets interconnected through mineral bridges have recently been shown to be a powerful approach to enhance both the strength and the toughness of nacre-like composites^16^. While previous examples of nacre-like transparent films and laminates^17,18^ or non-transparent glass-reinforced nacre-like composites^19^ have been reported, the combination of optical transparency with high strength and high fracture resistance in a bulk material remains an open challenge.

Here, we report a simple and scalable processing route to produce strong, tough, and transparent composites inspired by the structure of nacre. Combined high strength and toughness are achieved by using glass platelets that connect to each other via mineral bridges created during heat treatment of consolidated porous scaffolds. Infiltration of such interconnected scaffolds with a polymer matrix of same refractive index as glass results in nacre-like composites that are 2–3 times tougher than common glass and optically transparent, while reaching the strength values typical of soda-lime and silica glasses. The toughening effect observed is reflected in stable crack propagation during fracture and an increasing resistance against crack growth (rising R-curve behavior) that is very unusual in transparent brittle materials. In the following, we describe the simple workflow utilized to fabricate such composites and discuss their mechanical and optical properties in comparison with state-of-the-art transparent and strong structural glasses.

## Results

### Fabrication process

**Fig. 1:**
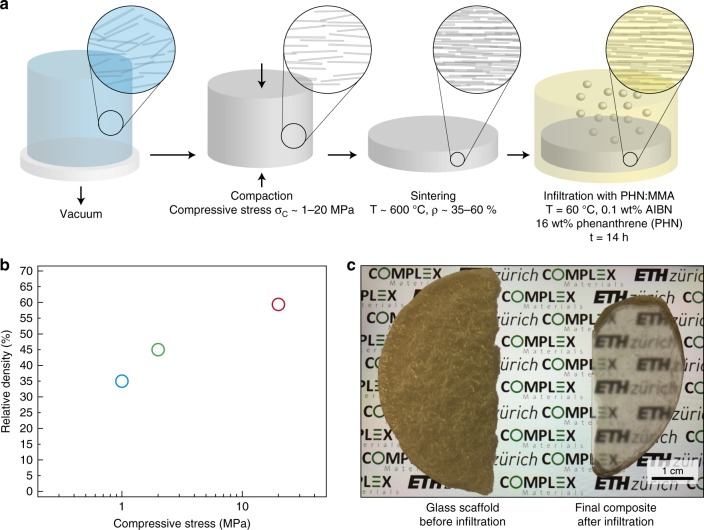
Overview of the transparent nacre-like composites. **a** Flow chart illustrating the preparation of transparent nacre-like composites. **b** Relative density of the final scaffolds after uniaxial compaction and sintering at 600 °C. Blue, green, and red symbols correspond to 35, 45, and 59% relative density, respectively. **c** Effect of the infiltration of a refractive-index matching polymer in a porous glass scaffold (left), resulting in a 2-mm-thick bioinspired transparent composite (right). Picture taken from a sample placed on top of a smartphone screen (retro-illuminated)

The fabrication of transparent bulk composites with a nacre-like architecture involves a series of simple filtration, compaction, and sintering steps, followed by the infiltration of the porous glass scaffold obtained with a refractive-index matching polymer (Fig. 1a). First, the glass flakes are dispersed in water and cast in a vacuum-aided filtration setup to form a green body. Due to their large size and aspect ratio of ~300, the glass flakes were first allowed to slowly sediment in the absence of vacuum in order to ensure their collective horizontal alignment, as already reported in similar systems^20,21^. Since the alignment and the assembly are based on the gravitational sedimentation of flakes with large size and aspect ratio, the thickness of the sample can be increased as high as needed by simply increasing the setup size and allowing the sedimentation of larger suspension volumes. This contrasts with the recently developed doctor-blading and paper-making approaches to fabricate nanoplatelet reinforced films and coatings^18,22^ that have reached only up to 200 -µm thicknesses. After the glass flakes have slowly settled on the filter paper, and the excess liquid displays optical clarity, vacuum is slowly applied to remove the excess water and form a green body with ~6 vol% of particles within 30–45 min. Similarly, the gravitational sedimentation within 1–3 days of ~30 aspect ratio alumina platelets has proved to be a viable strategy to fabricate bulk nacre-like green bodies (~cm thick)^20^, without, however, given the size of the particles and the viscosity of the liquid, being a time-effective alternative to our process. Water is still present in the green body, as it is transferred in a graphite mold and uniaxially compacted to a target specific mineral density. After compaction and drying, the green body is sintered at a temperature of 600 °C to create the mineral bridges at the contact points between the flakes, thus forming a porous glass scaffold. This sintering temperature is approximately half of that typically used in glass processing. Due to the presence of numerous scattering glass–air interfaces, the as-sintered glass scaffold behaves like a diffuse reflector and does not transmit light (Fig. 1c; Supplementary Fig. 1). In order to increase the optical transmittance, it is necessary to infiltrate the scaffold with a polymeric phase^23,24^ that has the same refractive index of the glass scaffold (*n*_glass_ = 1.52)^25^. We chose a mixture of poly(methyl methacrylate) (PMMA) and phenanthrene (PHN), as previous studies showed that 16% w/w PHN increases the refractive index of PMMA from 1.49 to 1.52^26,27^. After infiltration into the glass scaffolds at 60 °C, the methyl methacrylate:phenanthrene solution is bulk polymerized in situ following already-reported procedures^28–30^.

The density of the glass scaffolds after the sintering step can be tuned by varying the applied uniaxial compressive stresses within the range 1–20 MPa (Fig. 1b). Because of the low temperature used in the sintering step, the density of the scaffolds only marginally changes during the formation of the mineral bridges (Supplementary Fig. 2b). This allows us to precisely control the mineral content of the final nacre-like composite between 35 and 59%. Matching the refractive index of the mineral and the polymer phases leads to composites with significantly improved optical transmittance, as evidenced when the infiltrated glass scaffold is placed on top of a retro-illuminated pattern of a smartphone screen (Fig. 1c).

**Fig. 2:**
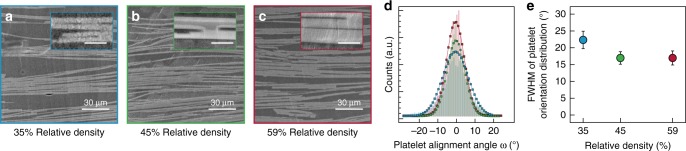
Microstructural analysis of the transparent and tough nacre-like composites. **a**–**c** Scanning electron micrographs of the polished cross sections of composites with relative densities of 35 (**a**), 45 (**b**), and 59% (**c**). Insets of **a**–**c** show details of the mineral bridges. Insets scale bars: 3 µm. **d** Distribution of alignment angles of the glass platelets in the three different microstructures. Gaussian distributions are used for fitting the data obtained using image analysis. **e** Full width at half maximum (FWHM) of the different angle distributions. At least ten images were used in the analysis of each scaffold density. The error bar represents the measured standard deviation

Vacuum-aided filtration followed by uniaxial compression are expected to collectively align the glass flakes horizontally in the green body in a nacre-inspired fashion. Since the alignment of flakes and the presence of mineral connections are crucial to obtain high mechanical properties and effective toughening mechanisms, these structural features were assessed through image analysis of samples at different mineral relative densities (Fig. 2).

### Composite microstructure

The microstructures of nacre-like composites with relative densities between 35 and 59% show strong alignment of platelets within the horizontal plane perpendicular to the compression axis (Fig. 2a–c). The application of higher compaction pressures does not disturb the preferential alignment and the aspect ratio of the glass platelets, which remain predominantly unbroken after compaction (Supplementary Fig. 3). The degree of platelet alignment was quantified by image analysis according to previously established methods^31^. The method proved to be a fast and convenient alternative to X-ray diffraction, which is not applicable to measure the alignment of our amorphous glass platelets. The platelet-alignment angles collected from the analysis of over ten micrographs for each mineral density were fitted with a Gaussian function (Fig. 2d). The degree of misalignment of the platelets in the micrographs can be quantified by the standard deviation obtained from the Gaussian fit, referred to here as the full width at half maximum (FWHM). This parameter was found to decrease from 23° to 17° when the relative density was increased from 35 to 45%. The FWHM values eventually saturate at 17° for higher mineral densities (Fig. 2e), which is similar to the levels of alignment achieved with more elaborated processing strategies^16,28,32^. Moreover, the alignment is preserved throughout the whole sample thickness (Supplementary Fig. 4). Our results demonstrate that slow sedimentation followed by a uniaxial compaction step at room temperature is an effective approach to achieve high levels of platelet alignment using a simple and scalable process. Such a process is expected to be particularly effective for flakes of larger dimensions and aspect ratio, as those utilized in this work.

The highly aligned platelets obtained after room-temperature mechanical compression can be interconnected via mineral bridges in a subsequent sintering process. The sintering step at 600 °C introduces mineral connections between individual platelets for all the composite mineral densities tested (insets Fig. 2a–c). Mineral bridges are formed due to the diffusion of ions within the contact points between glass platelets.

In addition to platelet alignment and the formation of mineral bridges, another structural feature observed in the nacre-like composites is the clustering of platelets. Such clusters lead to variations in the local density of the mineral phase within the sintered bodies at length scales on the order of tens of microns (Fig. 2a–c). The formation of clusters can be attributed to capillary forces exerted between adjacent platelets during the final drying of the green bodies, which tend to rearrange adjacent platelets into a stacked configuration that minimizes the air–water interfacial area. Such local inhomogeneity was found to be more pronounced in bodies with lower global relative density.

Despite the brittleness of both the glass flakes and phenanthrene-containing PMMA (PHN:PMMA), the alignment of the reinforcements and the presence of mineral connections should enhance the mechanical properties and the fracture resistance of the nacre-like composites by providing an interconnected stiff load-bearing phase and extrinsic toughening mechanisms against crack growth.

### Mechanical behavior

**Fig. 3:**
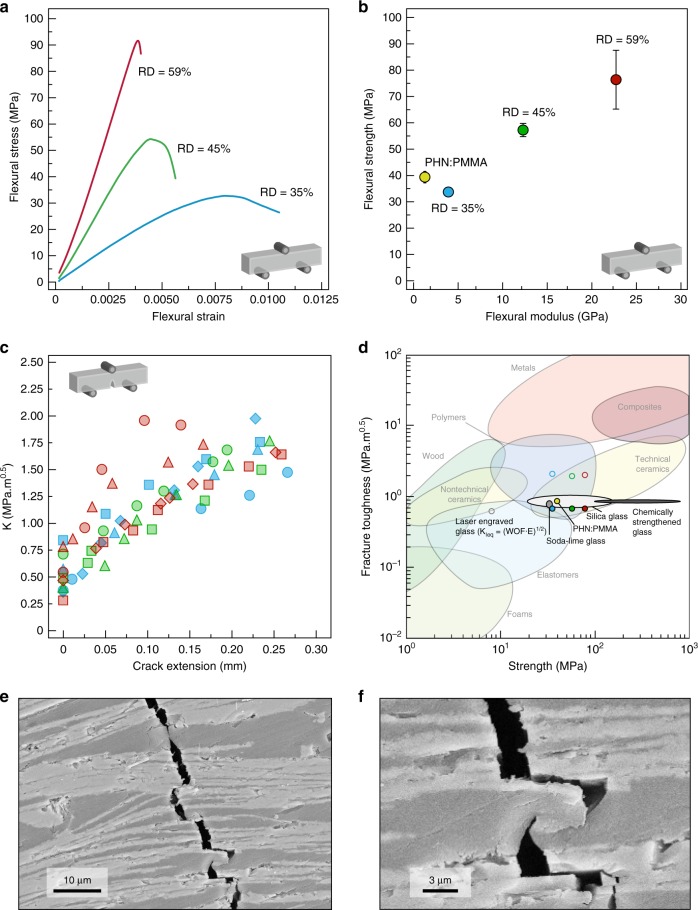
Mechanical characterization of the composites. **a** Flexural stress as a function of flexural strain for three composite beams with mineral contents of 35% (blue), 45% (green), and 59 vol% (red). **b** Flexural strength and elastic modulus of the composites and of the PHN:PMMA organic matrix (yellow tone). The error bar represents the measured standard deviation. **c** Crack growth resistance curves of the bulk nacre-like composites. Force-displacement curves are provided in Supplementary Fig. 5 along with a video of the crack propagation in a 35% dense specimen (Supplementary Movie 1). **d** Ashby plot of strength against fracture toughness for different classes of glass-based transparent materials. Our transparent nacre-like bulk composites, soda-lime glass^33^, silica glass^33^, borosilicate glass^34^, chemically toughened glass^35^, and laser engraved glass^34^ are highlighted. The data for the transparent nacre-like bulk composites are indicated by blue, green, and red circles, which correspond to specimens with 35, 45, and 59% relative density, respectively. Filled and empty circles represent K_IC_ and K_IJ max_, respectively. Adapted from ref. ^33^. **e**, **f** Scanning electron micrographs highlighting the fracture path within the composite (**e**) and a detail of the plastic deformation of the polymer (**f**)

To experimentally evaluate the mechanical properties of the nacre-like composites, we measured the Young’s modulus and the flexural strength in three-point bending of specimens with mineral content in the range 35–59 vol%. An increase of mineral content within this range enhances the elastic modulus and the fracture strength by fourfold and twofold, respectively (Fig. 3a, b). The absolute strength level of 75 MPa achieved by the composite with 59 vol% mineral phase matches the average strength values typical of borosilicate-^34^, silica- glasses^33^, and transparent polymers^33^ although the composites still contain a substantial fraction of a relatively weak polymer phase. This effect can be attributed to the microscale dimensions of the reinforcing glass phase used in our composites. Decreasing the size of stiff brittle materials often accounts for an improvement in their strength because of the reduction in critical defect sizes as the characteristic dimension of the material decreases^36,37^. In addition to this strengthening effect, the composites with mineral contents of 35 and 59 vol% reach elastic moduli that are, respectively, 2.6 and 11.7 times higher relative to the pure polymer phase. Due to the presence of the soft polymeric phase and the low amount of mineral bridges between the glass flakes, the elastic modulus of 23 GPa achieved with the highest mineral content is approximately one-third of the modulus of conventional glasses (~70 GPa). However, this difference drops to 2.3-fold if the specific elastic moduli of the materials are considered. Besides the flexural tests, we also measured the surface hardness of our material through conventional Vickers indentation (Supplementary Fig. 6). These results show that the achieved surface hardness is about ten times higher than any polymeric material, but still one order of magnitude lower than silica glass. With a tenfold higher hardness in comparison with the hardest polymers, the glass-based composites require ten times higher forces in order to generate visible scratches on their surface, which might be enough for several applications. Importantly, such scratches do not deteriorate the strength of the material as much as in harder silica glasses. The lower density of the composite also makes them an attractive low-weight alternative to conventional glasses. Therefore, the nacre-like glass-based materials offer a thus far inaccessible combination of hardness, low weight and damage tolerance.

The most remarkable mechanical feature of our nacre-like composites in comparison to state-of-the-art glass is their increasing resistance against fracture, also known as increasing R-curve behavior (Fig. 3c). The stress–strain curves obtained from the flexural tests already show the typical fingerprints of a graceful failure, with the stress decreasing gradually after reaching the maximum peak level (Fig. 3a). A graceful failure in brittle materials is usually associated with the presence of extrinsic toughening mechanisms that ensure the dissipation of the crack-driving force during fracture propagation.

The fracture behavior of our composites was examined in more details by performing single-edge notch-bending (SENB) tests and recording the crack propagation using a high-resolution camera. In these tests, the resistance of the material against crack growth is measured in terms of the stress intensity factor *K*_I_ as a crack propagates across the sample (Fig. 3c). The stress intensity factor required to initiate the crack (*K*_IC_) in our composites is comparable with the one of silica glass and 40% lower than the K_IC_ value of the pure PHN:PMMA polymer (Fig. 3d). The major difference to conventional glass and to the pure polymer is observed when the crack propagates within the material. In contrast to the typically constant *K*_IC_ values of standard glass and brittle PMMA, the crack growth resistance of our composites (*K*_IJ_) steadily increases as the crack extends further into the material. For crack extensions approaching 250 µm, the *K*_IJ_ values reach a maximum of 1.8 MPa m^0.5^ for all the investigated composites, which corresponds to a 2.5- to 3-fold increase in fracture toughness with respect to common glass and to the polymeric phase used in this study, PHN:PMMA (Fig. 3d). Our material effectively addresses the strength versus toughness conflict by introducing microscale toughening mechanisms in an otherwise stiff and brittle transparent material. To highlight this feature, we plotted our results in an Ashby map and compared our data to those of other transparent structural materials. The high fracture toughness achieved makes the nacre-like composites stand out in comparison with other state-of-the-art transparent materials (Fig. 3d). Whereas current glass-based materials show a clear trade-off between strength and toughness, the brick-and-mortar structure of our nacre-like composites opens the possibility of introducing flaw tolerance and improved toughness without significantly compromising the mechanical strength.

The rising R-curve observed for the nacre-like architectures reflects the interactions of the crack with the brick-and-mortar microstructure of the composite. Single-edge notched-bending tests on PHN:PMMA samples without the inorganic phase reveal the purely brittle behavior of the polymer phase (Supplementary Fig. 5b). This confirms that the R-curve behavior of the composites is solely due to the presence of extrinsic toughening mechanisms arising from the brick-and-mortar architecture. Scanning electron micrographs of the broken samples (Fig. 3e, f) show that toughening mechanisms involving crack deflection, crack bridging, and plastic deformation of the polymer clearly contribute to the rising R-curve behavior. Although this cannot be identified by image analysis, we expect the mismatch in Young’s modulus between the glass reinforcements and the PHN:PMMA matrix to also toughen the material by crack arresting mechanisms in the soft polymer phase^38,39^. To further elucidate the importance of a high toughness for a high-damage tolerance and hence high structural reliability, we used a bench driller to drill large holes in our composites. Not only the composites survived the drilling but the piece shows no extended damages from the holes (Supplementary Fig. 7).

**Fig. 4:**
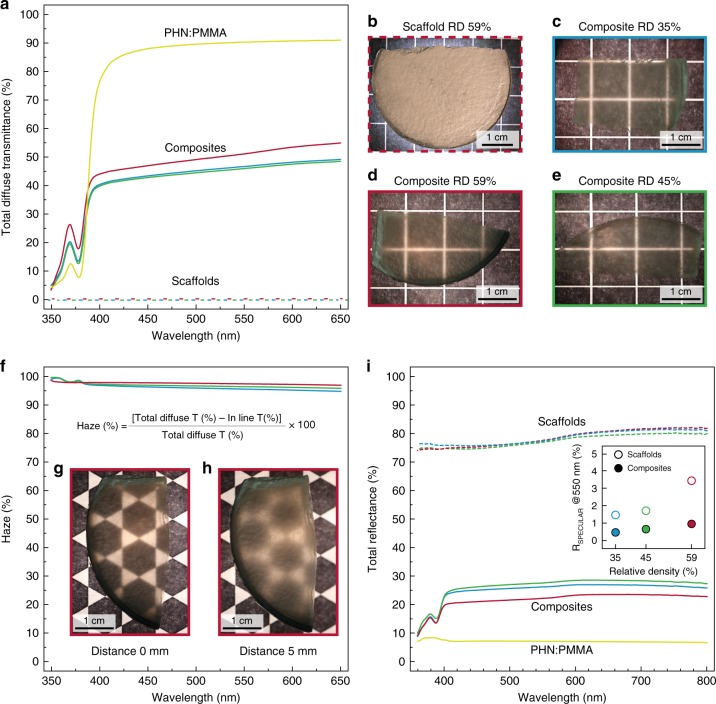
Optical characterization of the composites. **a** Total diffuse transmittance of glass scaffolds prior to infiltration (dashed lines) and of composites with mineral contents of 35% (blue), 45% (green), and 59% (red solid lines), respectively. The spectrum of PHN:PMMA is also displayed for comparison (solid yellow line). **b**–**e** Photographs of the porous scaffold (59% mineral content) and the infiltrated composites exhibiting clear increase in transmittance when the scaffold is infiltrated with the PHN:PMMA phase. Scale bars: 1 cm. All the pictures have been taken on top of a retro-illuminated pattern. **f** Haze, calculated as the ratio between diffuse transmittance and total diffuse transmittance. **g**, **h** Pictures of an infiltrated composite (59% mineral content) in contact (**g**) and 5 mm above (**h**) the retro-illuminated pattern. Scale bars: 1 cm. **i** Total reflectance measured on the scaffolds before infiltration (dashed lines), for the infiltrated composites (solid lines), and for the pure PHN:PMMA phase (solid yellow line). Specular reflectance of the composites as a function of mineral content measured at 550 nm (inset of **i**)

Besides the large increase in fracture toughness compared with state-of-the-art glass, our bioinspired architecture can also be designed to display a relatively high optical transparency and low reflectivity by matching the refractive indices of the polymer and the glass scaffold. To quantify the effect of polymer infiltration on the optical properties of the nacre-like composites relative the scaffold and the pure polymer, we optically characterized samples with different mineral contents in terms of the total diffuse transmittance of light, transmission haze, and total reflectance (Fig. 4).

### Optical properties

The total diffuse transmittance drastically improves from the non-infiltrated scaffolds to the infiltrated composites, regardless of their mineral content. For 1 -mm-thick samples, this is evidenced by the increase in transmittance T from ~0% up to values ranging from 45 to 55% across a broad range of the visible spectrum (Fig. 4a). A series of pictures taken on top of a simple background pattern highlights the relative difference in optical transmittance between the glass composites and the glass scaffolds (Fig. 4, b–e). The non-infiltrated nacre-like glass scaffold does not transmit light because it acts as a diffuse reflector (Fig. 4b), in which light is scattered at the many interfaces between the glass platelets and air. Strong scattering results from the large refractive-index (*n*) mismatch between glass and air: *n*_glass_ ≈ 1.52 and *n*_air_ ≈ 1.00. Infiltrating the glass scaffold with a refractive-index-matched polymer reduces scattering at these interfaces, thus decreasing the light dispersion and drastically increasing the transparency of the materials. Further increase in optical transparency beyond the reported values is still possible by reducing the thickness of the bulk material or optimizing the infiltration procedure to avoid the formation of air gaps within the polymer matrix. The composite surface roughness, measured to be in average 370 ± 3 nm, can also be decreased by adding a surface layer of pure polymer to improve the optical properties (see Supplementary Fig. 8). Since this will likely impact the hardness of the composite, a trade-off has to be found, depending on the specific application.

Despite the significant increase in the total diffuse transmittance, light is predominantly transmitted diffusely and not in-line. The diffused nature of the light transmitted through the nacre-like architectures leads to composites with high-transmission haze (Fig. 4f). Haze results from light scattering in all directions beyond the direction parallel to the incident beam (in-line). The reduction in the in-line transmittance due to scattering is also visible by the blurring effect observed when a composite slab is placed 5 mm above an illuminated background pattern. This is a macroscopic confirmation of the diffusive origin of large part of the transmitted light. We quantified the haze as the percentage of light diffused away from the incident direction over the total amount of transmitted light (see analytical formula in Fig. 4f). Our results show that the transmission haze is indeed close to 100% for all the investigated composites, regardless of their mineral content. As porosity is known to strongly influence the in-line transmittance and haze of transparent materials, we quantified the porosity and the pore-size distribution of our composites to theoretically estimate their effect on the optical properties of the nacre-like specimens. Mie scattering theory previously applied to ceramic materials with low porosity^40,41^ was used to estimate the theoretical in-line transmittance. With a measured porosity of 0.00073 and maximum radius of 0.065 µm (Supplementary Table 1), the theoretical in-line transmittance expected from the model is 6.8% (see Supplementary Discussion). A comparison between this estimated value and the measured in-line transmittance of 2% suggests that the air inclusions constitute a main source of in-line light attenuation and haze in the nacre-like composites. In addition to the presence of pores, the misalignment of the platelets (see Fig. 2) can translate into a broad range of refraction, reflection, and diffraction angles that can contribute to the diffuse transmittance of the specimens. Because of the haze effect, our nacre-like composites are most suitable as protective display covers that are placed close to the light source provided from the backscreen (Fig. 4g, h). In this scenario, the proximity to light sources, circuitry or other potential sources of heat may locally increase the temperature of the composite. Thermal analysis of samples with 59% relative density show that the composite can be reversibly cycled between 30 °C and 100 °C, without compromising its mechanical integrity (Supplementary Fig. 9). Moreover, the presence of mineral platelets reduces the coefficient of thermal expansion coefficient of the composite by one order of magnitude relative to the polymer, reaching values closer to those of pure glass. These low CTE values are important to minimize thermally induced interfacial stresses that can build up in case the composite is combined with glass in such display application. Alternatively, these composites can be utilized as glass-reinforced structural coatings in applications where the haze effect is desired.

Consistently with the higher light transmittance, the infiltration with a refractive-index-matched polymer lowers considerably the reflectance of the composites compared with the glass scaffolds. The total diffuse reflectance drops from around 75–80% to 20–25%, upon infiltration of the scaffolds with the PHN:PMMA polymer (Fig. 4i). The specular reflectance measured at 550 nm follows the same trend with a twofold lower value for the infiltrated scaffolds (inset Fig. 4i). Despite their low reflectance, the composites exhibit a nacre-like appearance that probably results from interference effects associated with a brick-and-mortar architecture (Supplementary Fig. 10).

## Discussion

In summary, bulk nacre-like composites combining optical transparency and rising crack growth resistance can be fabricated by infiltrating brick-and-mortar glass scaffolds with a refractive-index-matched organic phase. The brick-and-mortar architecture of the scaffold is easily created by slow sedimentation of high-aspect-ratio glass microplatelets followed by sintering at 600 °C to form mineral contacts between the inorganic platelets. This bioinspired structure leads to a crack growth resistance that is threefold higher than that of state-of-the-art chemically strengthened glass (Gorilla Glass 5), while keeping fracture strengths that are comparable with silica- and soda-lime glasses. Crack deflection, plastic deformation of the polymer phase and crack bridging were identified as the main mechanisms underlying the unusual toughness of these composites. Matching the refractive index of the glass and polymer phases results in up to 50% optical transmittance and 97% haze for 1-mm-thick slabs. By providing microstructural toughening mechanisms thus far inexistent in glass-based materials, the transparent nacre-like composites proposed here can be potentially used to replace or further reinforce structural glasses that combine high strength and optical transparency but suffer from low damage tolerance and brittle fracture, as is the case for protective display covers.

## Methods

### Scaffold fabrication

In total, 6.5 g of silica glass platelets with thickness of 1 µm and aspect ratio of 300 (ECR GlassFlake Unmilled, Grade GF100, Glassflake LTD, England) were suspended by vigorous stirring in 500 ml of DI water, leading to a 1 vol% suspension. The suspension was prepared without using additional dispersants to avoid carbon contamination during the subsequent heating treatment. The suspension of platelets was then transferred to a laboratory vacuum filtration setup. In this setup, a Büchner funnel (ø 45 mm) was extended with a Plexiglas tube to enable the filtering of up to 250 ml of liquid. The suspension (250 ml) was then poured into the tube and let slowly sediment on the filter paper for up to 15 min. The platelets were subsequently consolidated into a disc-shaped green body (ø 45 mm) by gently pulling vacuum for up to 30 min. The wet green body was loaded into a graphite dye (ø 50 mm) and, while still wet, further compacted in a uniaxial press at 0.5 mm/min speed (Instron, USA) at room temperature. Depending on the desired final density of the scaffold, the compression load was adjusted ranging from 1 to 20 kN. The wet and compacted green body was dried overnight to remove the excess of water. The compacted green body was sintered in an oven (Nabertherm GmbH, Germany) at 600 °C for 2 h. The obtained glass scaffolds were treated with a Piranha solution (3:1 H_2_SO_4_:H_2_O_2_) to prepare the surfaces of the glass platelets for the subsequent grafting treatment, and then washed thoroughly with water to remove the excess of solution. H_2_SO_4_ (sulfuric acid, puriss. 95.0–97.0, Sigma-Aldrich GmbH, Germany) and H_2_O_2_ (hydrogen peroxide, 35 wt%, ACROS ORGANICS GmbH, Germany) were used as received. The glass scaffolds were soaked up to 12 h into a 50:50 mixture of acetone anhydrous (VWR International S.A.S, France) and 3-(trimethoxysilyl)propyl methacrylate (gamma-MPS, Sigma-Aldrich GmbH, Germany), washed thoroughly with acetone, and finally dried. Before infiltration, a piece of the scaffold was cut using a wire saw (Well S.A., Switzerland) and its density characterized using the volumetric Archimedes measurement.

### Composite fabrication

A mixture of methyl methacrylate (MMA, Acros Organics GmbH, Germany), phenanthrene (PHN, abcr GmbH, Germany) and 2,2′-azobis(2–methylpropionitrile) (AIBN, Sigma-Aldrich GmbH, Germany) was prepared in a weight ratio of 1:0.16:0.001 and stirred vigorously to ease the dissolution of PHN in MMA. While stirring, the mixture was kept at 0 °C in order to avoid premature initiation reactions. The monomer solution was then degassed by bubbling N_2_ for up to 20 min in a Schlenk line. In the meantime, the sintered scaffolds were placed into 50 -ml centrifuge vessels, sealed with a rubber septum and kept under vacuum. Approximately 10–15 ml of monomer solution was injected in each of the centrifuge vessels. The monomer solution infiltrates the sintered part eased by the vacuum and produces bubbles, as the air in the pores gets replaced by the monomer solution. Approximately three cycles of N_2_ flushes, followed by gentle vacuum pulls, ensured the complete infiltration of the monomer solution in the porous scaffold, as the sintered scaffold stops bubbling. Finally, the reaction vessel was placed at 60 °C in an oil bath in order to activate the radical polymerization initiator (AIBN) while still keeping it under a slight overpressure of N_2_. The vessel was left at these conditions overnight (~14 h) to ensure complete polymerization. The following morning, the pieces were then post cured for 2 h at 100 °C in a vacuum oven (Thermo Fisher Scientific, USA), and kept under a N_2_ atmosphere. The composites were polished out of the excess embedding polymer using a rotating polishing machine (Struers GmbH, Germany) and prepared for the microstructural, optical, and mechanical characterization.

### Microstructural characterization

The relative alignment of the flakes in the cross-section of the sample was measured using the open-sourced image analysis software Fiji (*26*). The cross-sections were prepared for scanning electron imaging by first milling the surfaces using a broad ion beam milling system with Argon ions accelerated at 6 kV for 120 mins (Hitachi Ltd., Japan) and then by sputtering 3–6 nm of Pt on the surfaces in order to avoid charging effects during imaging. Finally, the cross-sections of the composites were imaged using a scanning electron microscope (SEM, LEO 1530, Zeiss GmbH, Germany). The porosity and the size distributions of the pores were measured using the open-sourced image analysis software Fiji (*26*) from high-resolution SEM cross-section images taken over a total area of 0.02 mm^2^. The identified pores were fitted using ellipses. Given the preferred orientation of the ellipses along the platelets alignment direction, the minor axis of the ellipses was chosen as representative of the pore size (Supplementary Fig. 11). Finally, the pore-size distribution was fitted using a lognormal function, leading to the maximum radius, *r*_*m*_, and standard deviation, σ (Supplementary Table 1).

### Surface roughness characterization

The roughness of the sample surface was measured with the help of the three-dimensional surface reconstruction tool of a Keyence microscope VHX5000 using a ×2000 magnification objective on areas of 0.25 mm^2^. In addition to optical measurements, atomic force microscopy (AFM) analysis was performed on a *WITec Atomic Force Microscope alpha300 A* in contact mode to further confirm the roughness analysis on a more local scale, covering an area of 0.025 mm^2^. In both cases, the arithmetical mean height (Sa) and the root mean-square height (Sq) were calculated according to the ISO 25178 (surface texture parameters) using Eq. (1) and Eq. (2):

1 $${\mathrm{Sa}} = \frac{1}{A}\mathop {\iint}\nolimits_{\hskip -6pt A} {\left| {Z(x,y)} \right|{\mathrm{dxdy}}}$$

2 $$S{\mathrm{q}} = \sqrt {\frac{1}{A}\mathop {\iint}\nolimits_{\hskip -6pt A} {{\mathrm{Z}}^2\left| {(x,y)} \right|{\mathrm{dxdy}}} }$$

The results of the measurements are summarized in Supplementary Table 2.

### Optical characterization

The optical characterization was performed using a UV–Vis spectrometer (Jasco Inc., USA) on 1–2-mm-thick samples. The measurements of the total diffuse transmittance of the composites were performed by equipping the spectrometer with an integrating sphere (150 mm Integrating Sphere, Model ILV-924, Jasco Inc., USA). The spectra were measured in absorbance and then normalized by dividing each spectrum by the thickness of the samples, and finally converting the data into transmittance values. The measurements of the in-line transmittance were performed at an angle of 0 ± 0.1° relative to the normal of the composite surface.

### Mechanical characterization

The composites were cut using a diamond saw (Struers GmbH, Germany). For single-edge notched bending (SENB), specimens of ~15 × 1.5 × 2 mm^3^ (*s*, *b*, *w*) were pre-notched using a 300 -µm diameter wire saw (Well S.A., Switzerland), and then manually sharpened using a razor blade. Before testing, the initial crack length *a* was measured using an optical microscope (Leica GmbH, Germany). The ratio *a*/*w* typically ranged from 0.4 to 0.45. The tests were performed on a Shimadzu AGS-X mechanical tester (Shimadzu Ltd., Japan) using a three-point bending setup with a span of 12 mm and a constant loading speed of 1 µm/s. The crack propagation was recorded on video by installing a camera with a resolution of 2.3 Mpx monitoring the evolution of the crack position during the fracture. The position and length of the crack extending parallel to the notch (mode I) across the whole sample thickness (Supplementary Fig. 12) was measured using Fiji, with an estimated error of around 8–10 µm. Given the overall straight trajectory of the crack, standard fracture mechanics analysis could be used to calculate the energy dissipated during crack propagation through the samples (*J*-integral) as a function of the crack extension^42–44^. The *J*-integral can be considered as the sum of an elastic and a plastic contribution (Eq. (3)):

3 $$J = J^{{\mathrm{EL}}} + J^{{\mathrm{PL}}}$$

The elastic part of the *J*-integral can be calculated from linear-elastic fracture mechanics theory, as displayed in Eq. (4):

4 $$J^{{\mathrm{EL}}} = \frac{{K_{\mathrm{I}}^2}}{{E{\prime} }}$$

with *K*_I_ being the stress intensity factor and *E*’ is the materials elastic modulus in conditions of plane strain, expressed by Eq. (5):

5 $$E{\prime} = \frac{E}{{(1 - {\mathrm{\upsilon }}^2)}}$$

In single-edge notched-bending tests, *K*_I_ can be calculated from the applied load *P*, as displayed in Eq. (6):

6 $$K_{\mathrm{I}} = \frac{{P \cdot s}}{{b \cdot w^{3/2}}} \cdot {\mathrm{f}}(a/w)$$

with *s*, *b*, and *w* being the span, lateral width, and thickness of the specimen. f(*a*/*w*) is a dimensionless function that can be calculated from Eq. (7):

7 $${\mathrm{f}}\left( {a/w} \right) = \frac{{3 \cdot (\frac{a}{w})^{1/2} \cdot \left[ {1.99 - (\frac{a}{w}) \cdot (1 - \frac{a}{w}) \cdot 2.15 - 3.93 \cdot \left( {\frac{a}{w}} \right) + 2.7 \cdot (\frac{a}{w})^2} \right]}}{{2 \cdot (1 + 2 \cdot \frac{a}{w}) \cdot (1 - \frac{a}{w})^{3/2}}}$$

The plastic part of the *J*-integral can be calculated using Eq. (8):

8 $$J^{{\mathrm{PL}}} = \frac{{1.9 \cdot A^{{\mathrm{PL}}}}}{{y \cdot b}}$$

with *A*^PL^ being the plastic area under the load-displacement curve and *y* the uncracked ligament length. As the uncracked ligament decreases with time due to crack propagation, the following corrected relation has been used (Eq. (9)):

9 $$J_{n}^{{\mathrm{PL}}} = \left[ {J_{{n} - 1}^{{\mathrm{PL}}} + \left(\frac{{1.9}}{{y_{{n} - 1}}}\right) \cdot \left(\frac{{{A}_{{n}}^{{\mathrm{PL}}} - A_{{{n}} - 1}^{{\mathrm{PL}}}}}{b}\right)} \right]\left[ {1 - \frac{{{a}_n - a_{n - 1}}}{{y_{n - 1}}}} \right]$$

To quantify both intrinsic and extrinsic toughening mechanisms, the stress intensity factor during crack propagation (*K*_I J_) was also calculated as reported in Eq. (10):

10 $$K_{{\mathrm{IJ}}} = \sqrt {(J^{{\mathrm{EL}}} + J^{{\mathrm{PL}}}) \cdot E{\prime} }$$

Beams with dimensions of 15 × 2 × 1.3 mm^3^ (*s*, *x*, *w*) containing glass platelets oriented in the *s*–*w* plane were cut and prepared for three-point bending flexural tests. The face in tension was mirror-polished and the edges beveled with a 4000 grit SiC grinding paper before testing. The tests were performed on a Shimadzu AGS-X mechanical tester (Shimadzu Ltd., Japan) using a three-point bending setup with a span of 12 mm and a constant loading speed of 1 µm/s. The tensile strength σ and strain *ε* values are calculated from the load *P* and beam deflection *d* in bending using Eq. (11) and Eq. (12):

11 $$\sigma = \frac{{3{\mathrm{PL}}}}{{2BD^2}}$$

and

12 $$\varepsilon = \frac{{6Dd}}{{L^2}}$$

where *B* and *D* are the width and thickness of the sample, respectively; and *L* is the testing span.

## Supplementary information

Supplementary Information

Supplementary Movie 1

Description of Additional Supplementary Files

## Data Availability

The data that support the conclusions presented in this paper are available from the corresponding author on request.
