# Supplementary material for: Transparent and tough bulk composites inspired by nacre
Source: Nat Commun. 2019 Jun 26;10:2794. doi: 10.1038/s41467-019-10829-2 (PMC6594953; doi:10.1038/s41467-019-10829-2)
Supplement: Supplementary file 1 — Supplementary Information [file 41467_2019_10829_MOESM1_ESM.pdf]

Transparent and tough bulk composites inspired by nacre

Magrini et al.

Supplementary Figures

a b

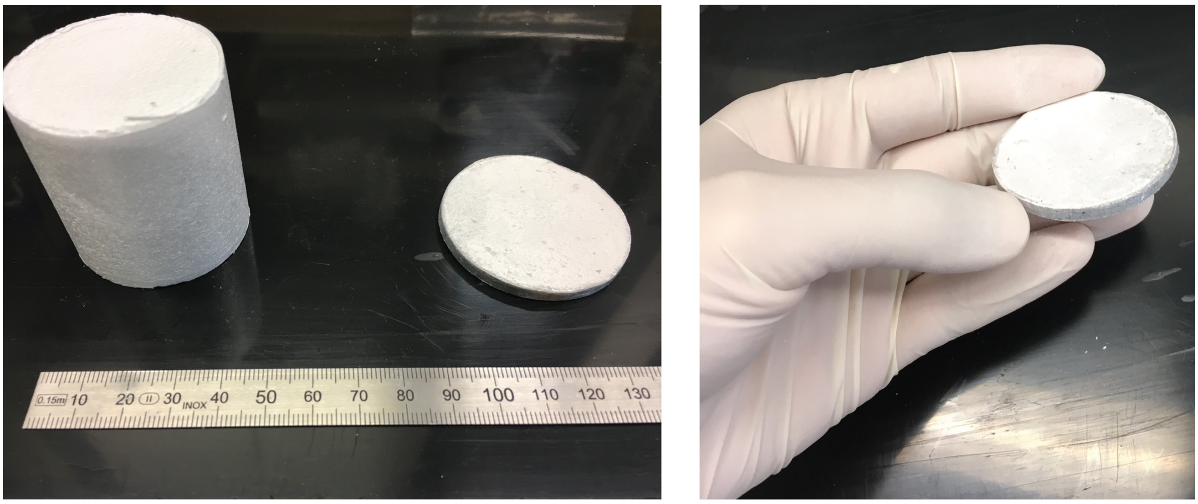

Supplementary Figure 1 | Evolution of sample size throughout the process. a Sample after sedimentation and filtering (left) and after pressing and sintering (right). **b** Side view of the sintered sample.

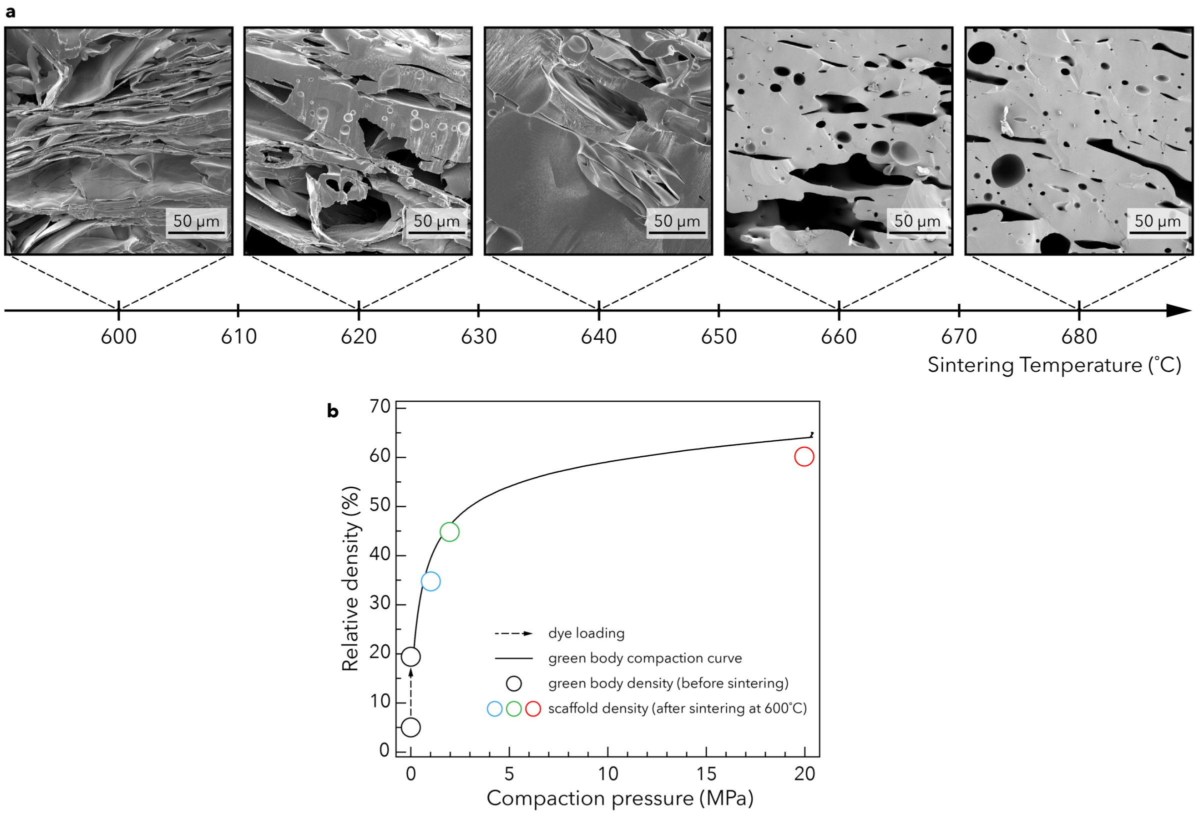

Supplementary Figure 2 | Effect of sintering temperature and compaction pressure on the nacre-like glass scaffolds. a Scanning electron micrographs showing the morphology changes of the glass scaffolds depending on the sintering temperature. **b** Compaction curve of a green body pressed up to 20 MPa in a graphite mold (black solid line). The symbols indicate the dry green body density (empty black symbol) and the final scaffold densities after sintering measured with the Archimedes method (empty colored symbols).

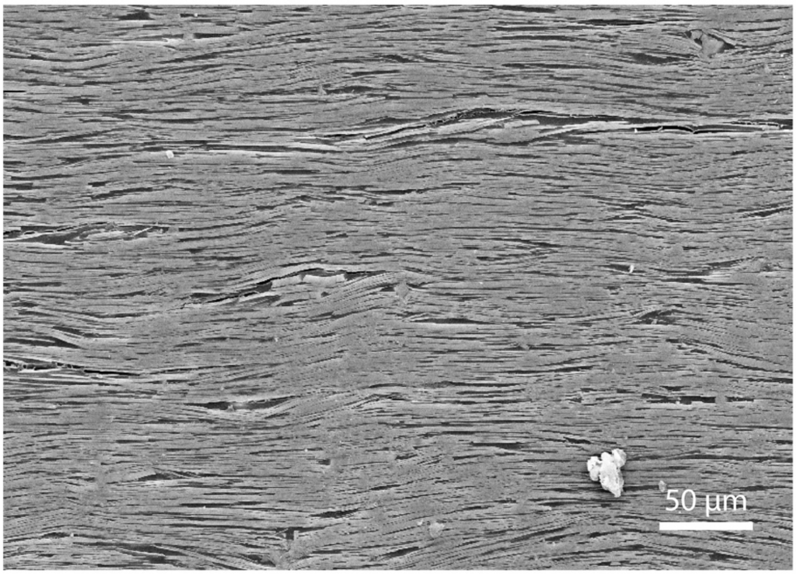

Supplementary Figure 3 | Microstructure of a sample subjected to high compaction pressure of 110 MPa. The scanning electron micrograph shows a highly oriented lamellar architecture with predominantly unbroken platelets.

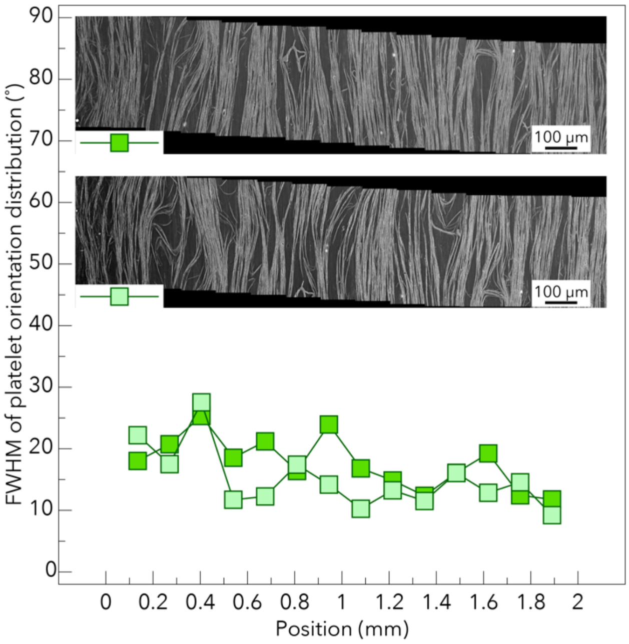

Supplementary Figure 4 | Evaluation of the platelets alignment throughout a 2-mm thick sample. Full width at half maximum (FWHM) of the platelet orientation angle distribution across the thickness of a sample having RD=45%. The distributions have been measured at two different positions.

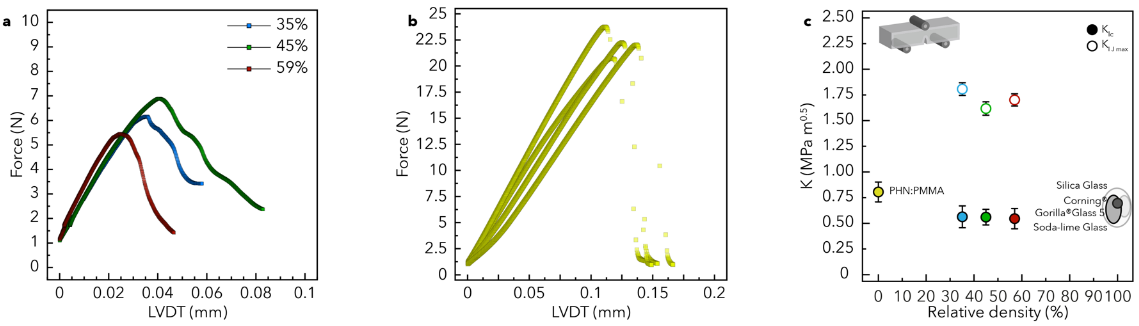

Supplementary Figure 5 | Fracture properties of the transparent nacre-like composite. a Force-displacement curves recorded during single edge notched bending (SENB) experiments. Composite specimens with mineral content of 35%, 45% and 59% are reported in blue, green and red, respectively. **b** Force-displacement curves recorded during single edge notched bending (SENB) experiments on the organic matrix PHN:PMMA. **c** Critical stress intensity factors for crack initiation (K_{IC}) (filled symbols) and crack propagation $K_{I\ Jmax}$ (empty symbols) measured for the different mineral contents. The error bars are representative of the standard deviation calculated over at least 3 samples per condition. Literature values from references[1,2].

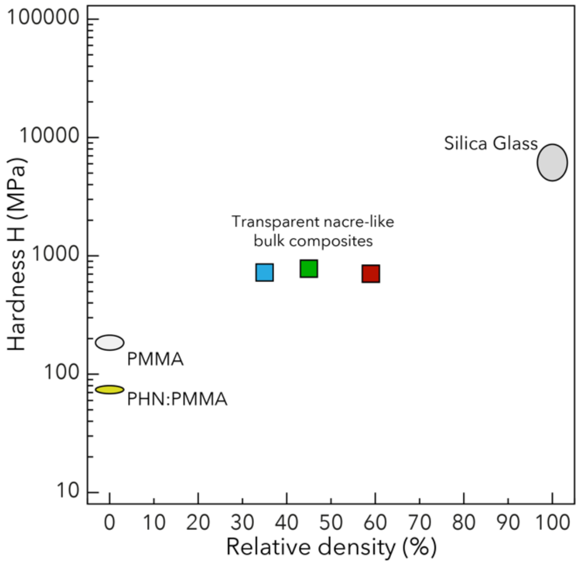

Supplementary Figure 6 | Surface hardness of the transparent nacre-like bulk composites. Vickers hardness values for the transparent nacre-like bulk composites and the organic phase PHN:PMMA measured with a load of 300 g and a loading time of 15s. As a comparison, the hardness values of PMMA and silica glass taken from literature[3] are reported as well.

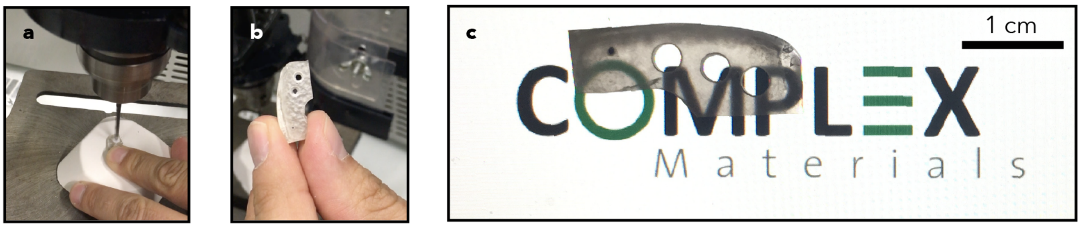

Supplementary Figure 7 | Damage tolerance of transparent nacre-like composites. a and **b** A bench driller is used to drill several holes, spaced approximately 1-1.2 mm apart, through the thickness of a transparent nacre-like bulk composite. **c** Picture of the still functional composite taken in contact with a retro-illuminated pattern (laptop screen).

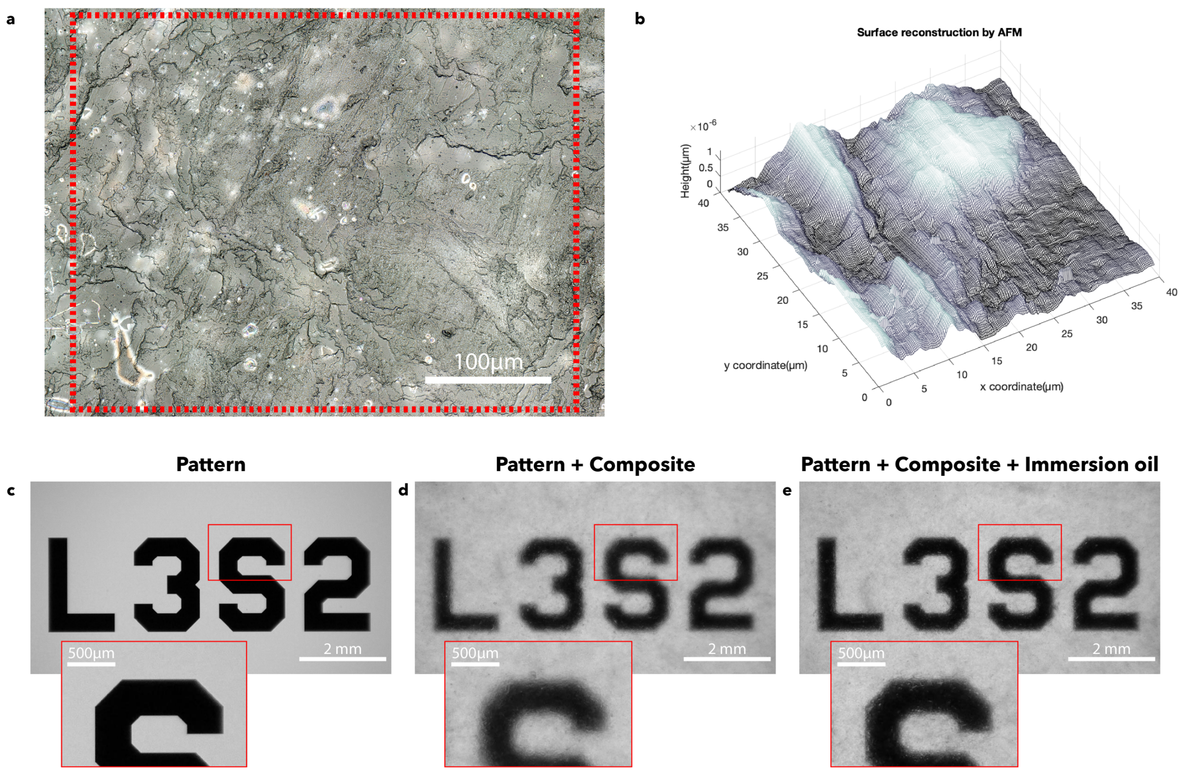

Supplementary Figure 8 | Surface roughness of the composites. **a** Reconstruction of the 3-dimensional topography of the surface of 45% dense transparent nacre-like composite from a large area stitch performed with an optical microscope. **b** Surface reconstruction of a smaller sample surface area performed using an Atomic Force Microscope (AFM). **c** to **e** Optical micrographs taken in retro-illumination highlighting the relative improvement from the as-polished specimen (**d**) to a specimen coupled to the substrate using a refractive index matching microscopy oil (**e**). **c** represents the pattern without any sample on. Insets of c to e show the relative improvement of a smaller detail of the pattern.

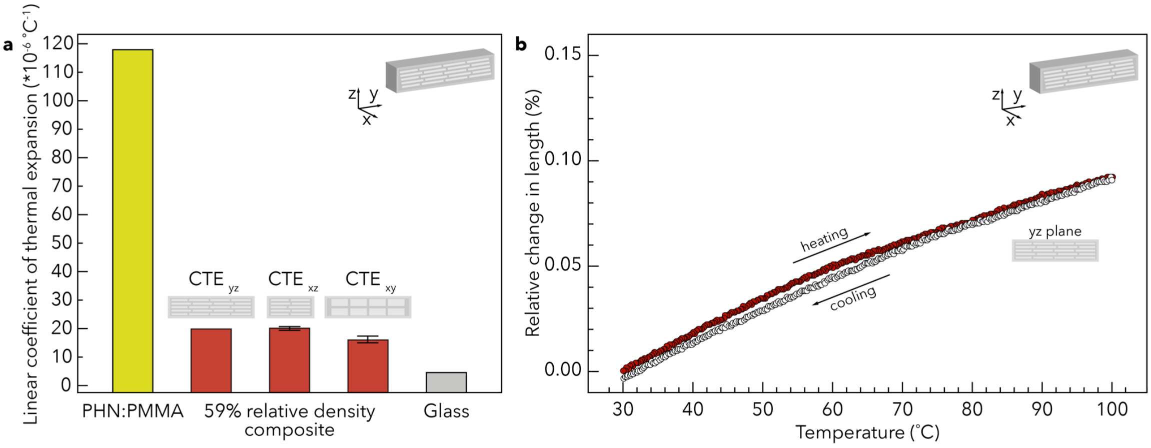

Supplementary Figure 9 | Thermal stability of the transparent nacre-like composites. a Linear coefficient of thermal expansion, α, of 59% RD specimens (red, measured), of the organic matrix PHN:PMMA (yellow, measured), and of glass[3]. The schematic representation of the specimen indicates the orientation of the measured planes relative to the platelet orientation. **b** Relative change in length as a function of temperature for the yz plane of a 59% RD composite during heating and subsequent cooling. The error bars are representative of the measured standard deviation.

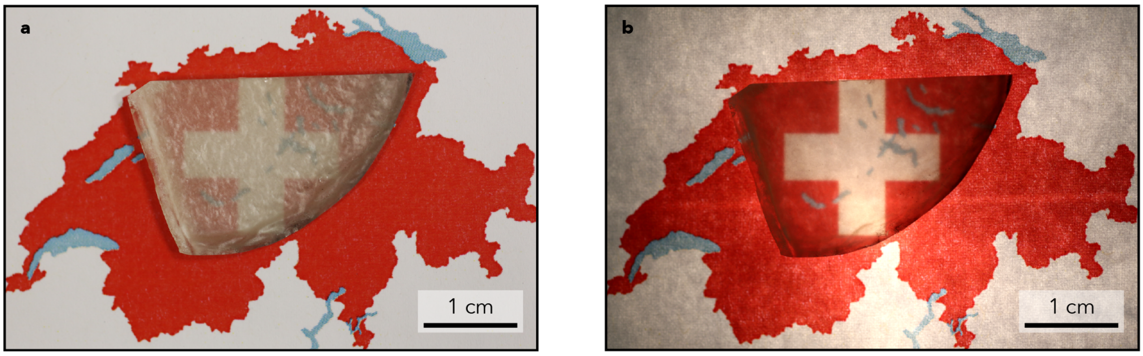

Supplementary Figure 10 | Relative difference in surface appearance of a 1.15-mm thick nacre-like composite illuminated from above and below. a Photograph taken in contact with a colored pattern illuminated from above, highlighting the nacre-like appearance of the object. **b** Photograph taken in contact with a colored pattern illuminated from below (retro-illuminated).

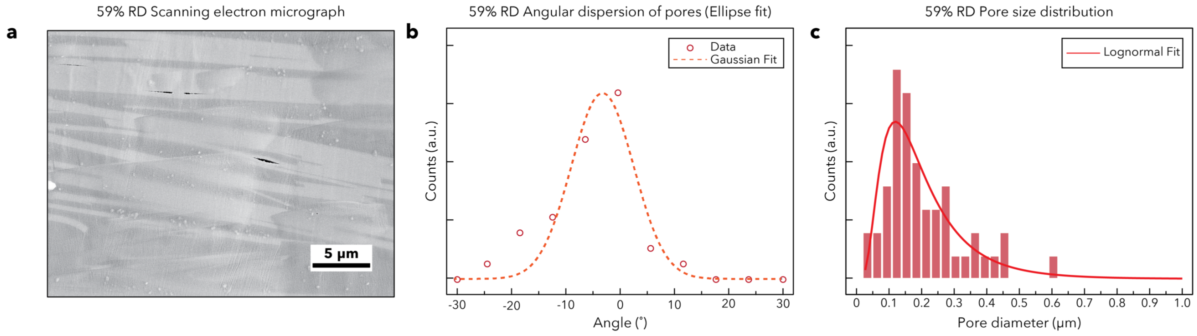

Supplementary Figure 11 | Size and orientation distribution or pores within the glass composites. a Scanning electron micrograph showing the elongated pores. **b** Angular distribution of the elliptical fits to the pores (scattered points). A Gaussian function was used for fitting (dashed red line). **c** Size distribution of the minor axis of the elliptical fits to the pores.

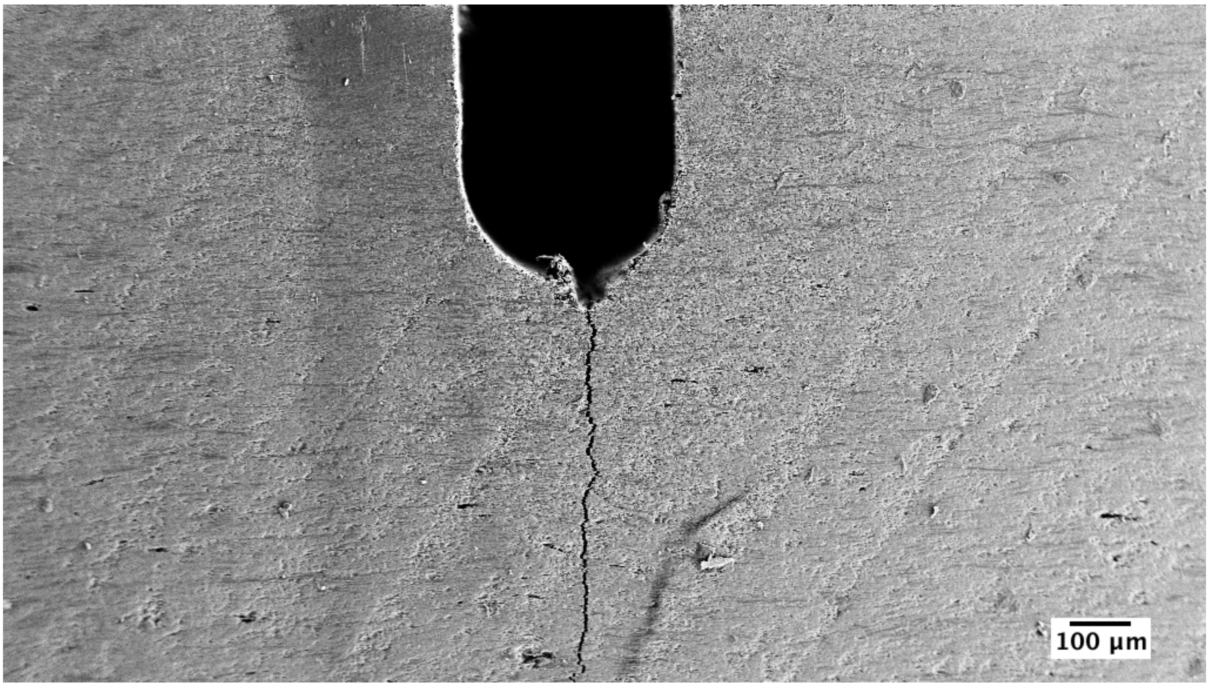

Supplementary Figure 12 | Crack trajectory in a SENB specimen. Scanning electron micrograph showing a typical crack path in a nacre-like composite with RD = 59%.

Supplementary Table 1 | Summary of the porosity P and the pore size distribution of nacre-like composites with relative density of 59%. The parameters r_m and σ represent the maximum radius and the standard deviation of the lognormal function used to fit the pore size distribution. The ILT_{th} value represents the theoretical estimate for the in-line transmission considering pores as the main scattering elements. ILT_{ex} is the measured in line transmittance of the composites.

Sample	Porosity P	r_m (μm)	σ	ILT_{th} (%)	ILT_{ex} (%)
Composite 59%RD	0.00073	0.065	0.6028	6.8 %	2.0 %

Supplementary Table 2 | Summary of the surface roughness measurements.

Method	Area of Interest (mm^2)	Sa (nm)	Sq (nm)
Optical Microscopy	0.25 ± 0.01	370 ± 30	500 ± 50
AFM	0.025	310 ± 13	377 ± 5

Supplementary Discussion

To evaluate the effect of porosity on the optical properties of the nacre-like composites, we have measured the areal porosity via image analysis on multiple cross-section high-resolution images taken with a scanning electron microscope. Measurements were performed on a total area of 0.02 mm^2, with a resolution of 25 nm/pixel. Using these images, we measured optically a total porosity P of 0.00073. In order to determine the pore size distribution, we applied a threshold and isolated several features per image. A threshold in the grey scale was easily identified due to the high contrast between the pores and the polymer. Given their shape, we chose to fit ellipses to the isolated features. The angular distribution of the fitted ellipses demonstrates that the pores are preferentially aligned parallel to each other and along the same direction as the glass flakes (Supplementary Figure 11 a and b).
The effect of the aligned porosity on the optical transmittance of the nacre-like composites was theoretically analysed with the help of previously proposed models based on Mie scattering theory. Assuming that the porosity is the main source of light attenuation in our composites, we applied in our analysis the scattering model proposed by Peelen and Metselaar for transparent alumina[4]. Alike our composites, transparent alumina shows a very low level of porosity. The theoretical in-line transmittance in such materials, ILT_{th}, is given by Supplementary Equation 1:

$$ILT_{th} = (1 - R)^2 exp(-C_{Sca} \cdot t) \qquad (1)$$

where R is the reflection at the two external surfaces of the sample, C_{Sca} is the scattering cross section and t is sample thickness.
The reflection R can be calculated using Supplementary Equation 2:

$$R = \frac{(m-1)^2}{(m+1)^2} \qquad (2)$$

where m is the refractive index of the composite, assumed to be 1.52 (@ 500 nm).
For spherical pores, C_{Sca} can be then calculated using the Supplementary Equation 3:

$$C_{Sca} = \frac{3}{4} \cdot \frac{P \cdot Q_{Eff}}{r_m \cdot \exp(3.5 \cdot \sigma^2)} \qquad (3)$$

where P is the porosity, Q_{Eff} is the effective scattering efficiency factor, and r_m and σ are the maximum radius and the standard deviation of a lognormal fitting to the pore size distribution, respectively.
For ellipse-shaped pores with major axes aligned perpendicular to the incident light, the scattering cross-section C_{Sca} can be calculated using the equation above if r_m and σ are taken from the distribution of the lengths of the minor axis of the elliptical fits (Supplementary Figure 11 c). Taking the experimentally measured values for P, r_m and σ (Supplementary Table 1), the only parameter that is still needed to estimate the in-line transmission (ILT_{th}) is the factor Q_{Eff}. This factor depends on the wavelength of the incident light and the refractive index of the solid phase. Taking an arbitrarily-chosen wavelength of 550 nm, we assume Q_{Eff}=1.1 based on previous numerical calculations performed for alumina.[4] While the scattering efficiency factor is expected to be different for the silica glass used in this study, we take this literature value for alumina as an approximation to be refined in further studies.
For a sample thickness of 1 mm and the measured porosity and pore size distribution shown in Supplementary Table 1, we estimate the theoretical in line transmittance (ILT_{th}) to be 6.8%. Considering that the experimental in-line transmittance (ILT_{ex}) at the same wavelength is 2.0%, our analysis suggests that light scattering by pores is a major contribution to the light attenuation observed in our nacre-like composites.

Supplementary References

1. Varshneya, A. K. in *Fundamentals of Inorganic Glasses* 409–453 (1994).

2. Varshneya, A. K. Chemical Strengthening of Glass: Lessons Learned and Yet To Be Learned. *Int. J. Appl. Glas. Sci.* **1,** 131–142 (2010).

3. Ashby, M. F. *Material Property Charts*. *Materials Selection in Mechanical Design* (Michael F. Ashby, 2011).

4. Peelen, J. G. J. & Metselaar, R. Light scattering by pores in polycrystalline materials: Transmission properties of alumina. *J. Appl. Phys.* **45,** 216–220 (1974).
