# Supplementary material for: Transparent and tough bulk composites inspired by nacre
Source: Nat Commun. 2019 Jun 26;10:2794. doi: 10.1038/s41467-019-10829-2 (PMC6594953; doi:10.1038/s41467-019-10829-2)
Supplement: Supplementary file 3 — Description of Additional Supplementary Files [file 41467_2019_10829_MOESM3_ESM.docx]

Description of Additional Supplementary Files

**Supplementary Movie 1:** Crack propagation during a single edge notch beam test of a transparent nacre-like composite.
